# Relationship Between Staphylococcus aureus Carriage and Surgical Site Infections Following Total Hip and Knee Arthroplasty in the South Asian Population: Protocol for a Prospective Cohort Study

**DOI:** 10.2196/10219

**Published:** 2018-06-06

**Authors:** Syed H Mufarrih, Nada Q Qureshi, Anum Sadruddin, Pervaiz Hashmi, Syed Faisal Mahmood, Afia Zafar, Shahryar Noordin

**Affiliations:** ^1^ Department of Orthopedic Surgery Aga Khan University Hospital Karachi Pakistan

**Keywords:** Staphylococcus aureus, Staph aureus, orthopedic surgery, surgical site infection, SSI, infections

## Abstract

**Background:**

Surgical site infections following total hip or knee arthroplasties have a reported rate of 0.49%-2.5% and can cause significant morbidity as well as tripling the cost of health care expenses. Both methicillin sensitive and methicillin resistant strains of *Staphylococcus aureus* surgical site infections have been established as a major risk factor for postoperative surgical site infections. *S. aureus* colonizes the nose, axillae, and perineal region in up to 20%-30% of individuals. Although the literature has reported a higher prevalence of methicillin resistant *S. aureus* in the South Asian population, routine preoperative screening and prophylaxis have not yet been implemented.

**Objective:**

The primary objective of our study is to identify the relationship between preoperative colonization status of *S. aureus* and incidence of postoperative surgical site infections in patients undergoing following total hip and knee arthroplasties. As part of the secondary objectives of this study, we will also investigate patient characteristics acting as risk factors for *S. aureus* colonization as well as the outcomes of total hip and knee arthroplasty patients which are affected by surgical site infections.

**Methods:**

This prospective cohort study will comprise of screening all patients older than 18 years of age admitted to the Aga Khan University Hospital for a primary total hip or knee arthroplasty for preoperative colonization with *S. aureus*. The patients will be followed postoperatively for up to one year following the surgery to assess the incidence of surgical site infections. The study duration will be 2 years (March 2018 to March 2020). For the purpose of screening, pooled swabs will be taken from the nose, axillae, and groin of each patient and inoculated in a brain heart infusion, followed by subculture onto mannitol salt agar and sheep blood agar. For methicillin resistant *S. aureus* identification, a cefoxitin disk screen will be done. Data will be analyzed using SPSS v23 and both univariate and multivariate regression analysis will be conducted.

**Results:**

Data collection for this study will commence at the Aga Khan University Hospital, Pakistan during March 2018.

**Conclusions:**

This study will not only estimate the true burden caused by *S. aureus* in the population under study but will also help identify the patients at a high risk of surgical site infections so that appropriate interventions, including prophylaxis with antibiotics such as muciprocin ointment or linezolid, can be made. Given the differences in lifestyle, quality, and affordability of health care and the geographical variation in patterns of antibiotic resistance, this study will contribute significantly to providing incentive for routine screening and prophylaxis for *S. aureus* including methicillin resistant *S. aureus* colonization in the South Asian population.

**Registered Report Identifier:**

RR1-10.2196/10219

## Introduction

### Background and Rationale

Although orthopedic surgeries are generally classified as clean, with strict aseptic techniques and antimicrobial prophylaxis commonly being employed, surgical site infections (SSIs) continue to be a critical complication. Several studies have reported high surgical site infection rates ranging from 0.49% up to 2.5% following hip and knee arthroplasties [1-8]. Although rare, the cost of an SSI to both the patient and the health care system is tremendous. By prolonging hospital stay by a median of 2 weeks, SSIs cause significant morbidity, and with the added rehospitalization costs, they can more than triple overall health care expenses [9].

Needless to say, the prevention of SSIs requires identification of risk factors with appropriate interventions [10]. *Staphylococcus aureus* is a major pathogen which colonizes the nose, axillae and perineal region in up to 20%-30% of individuals [11-15], with the most established risk factor for colonization being the extremes of age [16,17]. The association between *S. aureus* carrier status with an increased risk of *S. aureus* infections was first recorded by Danbolt in 1931 [18]. It has been shown that carriers of *S. aureus* are 2 to 9 times more likely to acquire *S. aureus* SSIs than noncarriers [19-21]. In fact, a study has shown that nasal carriage was the only independent risk factor found for *S. aureus* SSIs in patients undergoing orthopedic implant surgery [5]. Furthermore, in patients who acquire *S. aureus* SSIs, paired *S. aureus* isolates from the wound match those from the nares 85% of the time [22]. Additionally, and more importantly, methicillin resistant *Staphylococcus aureus* (MRSA), a variant of *S. aureus* which is becoming an increasingly common pathogen, constitutes 2.58% of *S. aureus* isolates. MRSA colonization has been found to be associated with worse clinical outcomes after surgery and higher rates of mortality [23].

It has been reported previously that preoperative identification and decolonization with muciprocin ointment decreased the risk of staphylococcal infections from 2.6% to 1.5%. In addition to this, the number of non-*staphylococcal* infections is also decreased [24].

Knowledge of geographical variation in antibiotic resistance patterns is not new. Studies have reported a higher prevalence of MRSA in South Asian countries, approximately as high as 10.7%-19.51% [25-27]. However, the results of our hospital data from the past two years show a total of 3 SSIs following knee arthroplasty, none of which were caused by *S. aureus* [28]. This data suggests that *S. aureus* may not be the priority concern when it comes to SSIs following knee arthroplasties in our population. Given the resource limitation and fee for service policy in this part of the world, it is best to focus prophylaxis on the organisms shown to be responsible for SSIs in our data, such as gram negative bacteria. Therefore, this study will play a role in establishing whether *S. aureus* is truly a major concern in this population and whether it is cost effective to make it the primary target for prophylactic therapies. The aim of our study is to estimate the incidence of SSIs caused by *S. aureus* and the prevalence of methicillin sensitive *Staphylococcus aureus* (MSSA) and/or MRSA colonization in the population presenting for elective orthopedic surgeries. To our knowledge, similar studies have not been conducted in the South Asian population and we hope that the results from our study will allow for appropriate interventions to be sought and implemented to promote superior health care practices for all.

### Objectives

#### Primary Objective

The primary objective of this study is to determine the relationship between preoperative colonization of the nose, axillae, and groin by MSSA and/or MRSA and postoperative SSIs by MSSA and/or MRSA following elective total knee or hip arthroplasties.

#### Secondary Objectives

The secondary objectives of this study are listed below.

1. To estimate the incidence of surgical site infections caused by MSSA and/or MRSA following elective total hip or knee arthroplasty.
2. To identify patient characteristics associated with MSSA and/or MRSA colonization.
3. To evaluate the outcomes of total hip or knee arthroplasty in patients who are preoperatively colonized with *S. aureus* and postoperatively have an SSI with MRSA and/or MSSA.

## Methods

### Study Design

In this prospective cohort study, over the course of a year, patients admitted to a single tertiary care hospital for an elective total hip or knee arthroplasty will be screened for preoperative colonization with *S. aureus* (MRSA and/or MSSA). Postoperative follow-ups with these patients will then occur for up to one year to assess and document SSIs, among other outcome variables.

### Setting

The study will be conducted at the Aga Khan University in Pakistan over a period of 2 years beginning March 2018. The expected completion date of the study is March 2020. Screening will be conducted for all patients admitted during the first year. The next year will be utilized to complete the one year of follow-up of each patient.

### Participants

#### Inclusion Criteria

The inclusion criteria for participants in this study are as follows:

- All patients admitted for an elective total hip arthroplasty.
- All patients admitted for an elective total knee arthroplasty.
- Patients older than 18 years of age.

#### Exclusion Criteria

The exclusion criteria for participants in this study are as follows:

- Patients with history of MRSA infections in the last one month (based on culture positivity or having received treatment for MRSA).
- Patients undergoing revision arthroplasty.

### Sample Size

We used Sample Size Determination in Health Studies (Version 2.0, 1998, WHO) to apply a formula for hypothesis testing using relative risk in cohort studies. In a previous study, Prince et al reported that the rate of infection in colonized groups is 4.7%, while it is 1% in noncolonized groups [15]. Thus, assuming the rate of infection in the colonized group is 4.7% versus 1% in the noncolonized group, we take the value of the relative risk due to colonization to be 4.7% with a level of significance at 5% and power of 0.9. This calculation showed the need for a minimum sample size of 423 subjects. Therefore, we intend to recruit 500 subjects to account for the losses in follow-up.

### Variables

Both independent and dependent variables, as well as potential confounders, will be recorded in this study. The independent variable in this study is the carrier status of the patient, either positive (carrier of MRSA or MSSA or both) or negative.

The dependent variables in this study are as follows: surgical site status (infected or not), postoperative length of hospital stay (prolonged or not), postoperative complications (yes or no), and rehospitalizations due to surgical site infection (yes or no). A prolonged hospital stay will be defined as >1 SD from the mean hospital stay calculated for total hip or knee arthroplasties in our sample.

Potential confounders identified in this study are as follows: patient’s sex (male or female), age at operation (≥65 years or <65 years), body mass index (≥30 kg/m^2^ or <30k g/m^2^), comorbid conditions (yes or no), type of procedure (hip vs knee), duration of surgery (prolonged vs non prolonged), American Society of Anaesthesiologist’s status (≥3 or <3), previous hospital admissions within 6 months (yes or no), and antibiotic therapy within one month of current admission (yes or no). A prolonged duration of surgery will be defined as >1 SD from the mean duration of surgery calculated for total hip or knee arthroplasties in our sample.

### Data Sources and Collection

Patients eligible to participate in the study as per the inclusion and exclusion criteria will be approached by 2 trained researchers, 1 male and 1 female, at their respective beds in the wards on the day of admission, prior to the commencement of standard preparation for surgery. The researchers will explain the purpose and procedure to them in the local language and obtain consent. They will provide a written consent form (Multimedia Appendix 1) which the patients will be asked to sign if they accept the invitation to participate in the study. If any patient is unable to consent for themselves for reasons including, but not limited to, cognitive impairment or physical incapacitation, consent will be obtained from the health care proxy as applicable. During this first interaction with the patient, samples will be taken and sent to the microbiology lab to determine colonization, if any, by *S. aureus* (details of sample collection are described below) and all questions pertaining to the patient’s demographics and preoperative characteristics will be recorded in the questionnaire (Multimedia Appendix 2). The patient will undergo the planned surgery the following day. Postoperatively, the patient’s medical record files will be used to document the details of the surgical variables relevant to this study. As per guidelines for arthroplasties, all patients receive a cefazolin dose intraoperatively and 3 doses postoperatively. Any variations in this regimen will be noted and included in the analysis.

#### Patient Follow-up

Follow-ups of patients included in this study will be conducted via phone calls made by the research officer at 2 weeks, 2 months, 3 months, 6 months, and 1 year after discharge from the hospital stay following the initial surgery. If any symptoms reported by the patients are suspicious, they will be advised to visit their primary physician for a follow-up where documentation of an SSI will occur using the criteria listed below. Following examination, if an infection is suspected, attending surgeons will be encouraged to send samples from the surgical site to culture so identification of MSSA and/or MRSA can be conducted. Patients lost to follow-up will be excluded from the analysis.

#### Study Outcome Definition

Diagnosis of an SSI will be based on the criteria put forward by the Centers for Disease Control and Prevention [10]. An SSI will be classified into one of 3 groups listed below.

1. Superficial incisional surgical site infection occurring within 30 days of surgery.
2. Deep incisional surgical site infection.
3. Organ or space surgical site infections occurring within 30 days of surgery if no implant is left in place or within 1 year if the implant was in place and the infection appeared to be related to the surgery.

All pathogens will be examined for all SSI cases. An SSI with MRSA will be assessed using the same culture method as used for assessment of nasal MRSA.

#### Sample Collection

For the purpose of screening for MRSA or MSSA, pooled swabs will be taken from the nose, axillae, and groin of each patient. A total of 3 transport swabs will be used for each patient, one for both nares, one for both axillae, and one for the bilateral groin region. For adequate sampling, each swab will be rubbed for a total of 5-6 seconds in each region. Three transport swabs for each patient will be labelled with a single code and transported to the microbiology lab for further processing.

#### Laboratory Procedure

Pooled swabs from each patient will be inoculated in a brain heart infusion for 24 hours at 37⁰C, following which the specimen will be subcultured onto mannitol salt agar and sheep blood agar. The agar plates will be assessed after 24 hours. For none or minimal growth, the plate will be reincubated and reassessed at 48 hours. *S. aureus* will be identified by tube coagulase and deoxyribonucleic acidase production. For MRSA identification, a cefoxitin disk screen will be conducted.

### Statistical Analysis

Patients admitted several times during the study period will be included only once in the analysis. Data will be analyzed using SPSS v23. The Shapiro-Wilk Test will be used to access normality of the variables. For normally distributed data, means will be reported and comparison will be done using a *t* test or Wilcoxon signed rank test. For skewed data, medians with interquartile ranges will be reported and comparisons will be conducted using the Mann-Whitney *U* test. For categorical variables, we will use a chi-square test for comparison. If chi square assumptions are violated, the Fisher exact test will be used. In addition, a Kaplan-Meier survival analysis will be used to compare the two groups; the patients who were tested positive for MRSA or MSSA colonization and patients who were negative for MRSA or MSSA colonization.

Multiple logistic regression analysis will be performed to estimate adjusted relative risk (odds ratios, ORs) and their 95% CIs. For univariate testing, the threshold for qualifying for further analysis will be *P* value <0.20. All variables with *P* values <0.05 in multivariate regression analysis will be declared significant. The test for trend will be performed by including explanatory variables in the model that will be coded by ordinal numbers with increasing categories of exposure.

### Ethical Approval

Approval for the conduction of this study has been taken from the Ethical Review Committee (ERC) of Aga Khan University Hospital, Pakistan; ERC Number: 4014-Sur-ERC-16.

## Results

Data collection for this study will commence at the Aga Khan University Hospital, Pakistan, on March 5, 2018.

As part of the primary objective and secondary objective three, we will demonstrate the relationship between the preoperative carrier status (independent variable) and postoperative SSI (dependent variable for the primary objective) and other dependent variables as a crude and adjusted relative risk (RR), as shown in example Multimedia Appendix 3.

As part of secondary objective two, we will demonstrate the relationship between the carrier status (independent variable) and potential confounding variables as both crude and adjusted ORs, as shown in example Multimedia Appendix 4.

## Discussion

### Principal Results

The South Asian population belongs to the developing world. Together with the differences in lifestyle, quality of health care, and the affordability of health care expenses, the geographical variation in patterns of antibiotic resistance makes it imperative to study the incidence of SSIs caused by *S. aureus*, particularly MRSA, with emphasis on the relationship between preoperative colonization and postoperative infections. This will not only help to identify the patients at a high risk of SSIs, but also allow the staging of appropriate interventions, such as prophylaxis with an antibiotic which provides MRSA coverage (eg, muciprocin ointment).

Although no study investigating the relationship between preoperative colonization and postoperative infection by *S. aureus* for orthopedic surgeries has been conducted in our region where the aseptic measures may be stricter, similar studies in other regions have been conducted in the past. In 2001, Ziaullah et al reported that out of 308 hospital personnel, 20.8% were found to be nasal carriers of *S. aureus*, with MRSA accounting for 10.7% of the samples [27]. In 2014, Anwar and colleagues reported that out of 1660 nasal samples taken from patients’ attendants, a total of 246 (14.82%) samples were positive for growth of *S. aureus*. Out of the 246 positive samples, 48 (19.51%) isolates were MRSA [26]. In the same year, Khurram et al reported that out of 1431 patients admitted in the ICU, 57 patients developed infections with MRSA. They showed that older (>52 years), diabetic patients with a central venous line in place were at a significantly higher risk of developing these infections [29]. The report also showed that the rate of surgical site infection following clean cardiovascular surgery was 4%; 40% of which was caused by *S. aureus* [29].

Our study aims to investigate the relationship between preoperative colonization and postoperative infections by *S. aureus*. Although this study does justice to the defined objectives within the scope of resources available to us, the elaboration of certain issues and relevant recommendations may help in drawing stronger conclusions from future studies.

The methodology described will accurately depict a correlation between preoperative carrier status and postoperative infection by *S. aureus.* However, in order to establish a causal relationship between the two variables, the *S. aureus* strains need to be typed (eg, by pulse field gel electrophoresis) [30]. The recommendations to administer antibiotic prophylaxis with MRSA coverage could nevertheless still be made if the incidence of postoperative MRSA infections was found to be significant. In addition to preoperative cultures, intraoperative and postoperative cultures, together with cultures at regular follow-up intervals, can be planned for future studies to account for acquisition of *S. aureus* during and after the surgical procedure.

In our study, we are using pooled swabs from the nose, axillae, and groin cultured together to establish the carrier status. Although culturing the sample from each region separately would increase the cost, it would allow us to identify the most common area for colonization by *S. aureus*. This may impact the choice of agent to be used as antibiotic prophylaxis since muciprocin ointment applied locally for intranasal colonization has been a popular choice, but its application over large areas increases its resistance [17]. So, if the axillae and groin were also found to be colonized, systemic antibiotics such as linezolid could be considered as an alternative [31]. Furthermore, quantification of culture results could also aid in the making the decision between local and systemic antibiotics.

The methodology described in our study for the follow-up of patients up to one year postsurgery has some limitations. Firstly, patients may acquire MRSA from subsequent hospitalizations other than those in our hospital following discharge and may be prescribed antibiotics for infections other than those of the surgical site during the one year of follow-up. Secondly, a long follow-up adds to the potential confounders which may influence the results. Therefore, we recommend a more throrough documentation of potential confounding variables and an elaborate analysis plan to take those factors into account to draw more robust conclusions from future studies.

### Benefits and Potential Risks

The treatment plan for all patients will follow the standard protocol regardless of participation in the study. Specimen sample collection itself will take a maximum of 15 minutes of the patient’s time if they choose to participate. Results of a positive screening for MSSA or MRSA will be communicated to the patient via telephone, so they can use this information in any future surgeries they undergo.

### Conclusion

This prospective cohort study will add to the current literature by investigating the relationship between preoperative colonization and the postoperative incidence of SSIs by MSSA and/or MRSA following orthopedic surgeries in the South Asian population. The study will allow for the identification of patients at a higher risk of developing an SSI so that appropriate interventions including local or systemic antibiotic prophylaxis can be planned. This may lead to a reduction in the rates of SSIs following relatively expensive surgeries and decreasing hospital costs for a population which belongs to the developing world.

## Appendix

Multimedia Appendix 1 Consent form.

Multimedia Appendix 2 Demographics and preoperative characteristics questionnaire.

Multimedia Appendix 3 Relative risk of each dependent variable with a positive carrier status for *S. aureus*.

Multimedia Appendix 4 The relationship between a positive carrier status of *S. aureus* and various patient characteristics.

## Abbreviations

ASA: American Society of Anesthesiologists
ERC: Ethical Review Committee
MRSA: Methicillin resistant *Staphylococcus aureus*
MSSA: Methicillin sensitive *Staphylococcus aureus*
OR: odds ratio
RR: relative risk
SSI: surgical site infection
