# Supplementary material for: Relationship Between Staphylococcus aureus Carriage and Surgical Site Infections Following Total Hip and Knee Arthroplasty in the South Asian Population: Protocol for a Prospective Cohort Study
Source: JMIR Res Protoc. 2018 Jun 6;7(6):e10219. doi: 10.2196/10219 (PMC6283255; doi:10.2196/10219)
Supplement: Multimedia Appendix 1 [file resprot_v7i6e10219_app1.pdf]

STAPHYLOCOCCUS AUREUS CARRIAGE AND SURGICAL-SITE INFECTIONS IN ORTHOPEDIC SURGERY

Title of study: Staphylococcus aureus carriage and surgical-site infections in Orthopedic Surgery

Principal investigator: Dr. Shahryar Noordin, Dr. Pervaiz Hashmi, Dr. Faisal Mahmood & Dr. Afia Zafar

Institute: Section of Orthopedics, Department of Surgery, Aga Khan University

Introduction:

I am Dr. ______________________________ from Section of orthopedics, Department of surgery, The Aga Khan University and doing a study on staphylococcus aureus carriage and surgical-site infections (SSI) in orthopedic surgery. I want to see what factors are associated with carriage of this organism and its relation to surgical-site infections in patients who are managed at Aga Khan University, section of Orthopedic Surgery. I would like you to join this research study.

Background information:

There is a strong epidemiologic association between nasal carriage of S. aureus and development of S. aureus surgical-site infections. Carriers are two to nine times more likely to acquire S. aureus SSIs than noncarriers. Infact, nasal carriage has been shown to be the only independent risk factor for S. aureus SSI in patients undergoing orthopaedic implant surgery. Furthermore, in patients who acquire S. aureus SSIs, paired S. aureus isolates from the wound match those from the nares 85% of the time.

Purpose of this research study.

This study has been designed to determine the relationship between the existence of MRSA in the nasal cavity, groin and axillae preoperatively and the occurrence of surgical site infections with MRSA after orthopedic surgery. The primary outcome of the study will be the cumulative incidence of nosocomial S. aureus infections

Elucidating the mechanisms involved in surgical site infections will help formulate preventive strategies for this problem. Accordingly, reduced surgical site infections would translate into enhanced quality of care in addition to economic savings for the patient as well as the health care system.

Procedures

Patients admitted to Aga Khan University Hospital, Karachi for elective hip or knee replacement, proximal femur fractures including intertrochanteric / sub-trochanteric and femoral neck fractures will be enrolled in the study. A pooled specimen swab from the nares, groin and axillae will be taken preoperatively to check for the presence of MRSA. Patients will be followed up for atleast one year after surgery via phone call in order to assess whether they developed SSI with MRSA or other pathogens. Data will be analyzed on SPSS.

Possible risks or benefits

There is no risk involved in this study except your valuable time. There is no direct benefit to you also. However, the results of the study may help us to formulate primary prevention strategies for this significant morbidity that has a devastating potential. On the other hand, for patients requiring surgery, this study will provide us with precise data regarding surgical site infections which is an important benchmark with respect to international accreditation systems.

Right of refusal to participate and withdrawal

You are free to choose to participate in the study. You may refuse to participate without any loss of benefit which you are otherwise entitled to. You will receive the same standard care and treatment which is considered best for you irrespective of your participation in the study. You may also withdraw any time from the study without any adverse effect on your management or any loss of benefit which you are otherwise entitled to. You may also refuse to answer some or all the questions if you don't feel comfortable with those questions.

Confidentiality

The information provided by you will remain confidential. Nobody except principal investigator will have an access to it. Your name and identity will also not be disclosed at any time. However the data may be seen by Ethical Review Committee and may be published in journal and elsewhere without giving your name or disclosing your identity.

Available Sources of Information

If you have any further questions you may contact Principal Investigator (Dr. Shahryar Noordin), section of Orthopedics, Department of Surgery at Aga Khan University on following phone number 0092-21-3486-4384.

1. AUTHORIZATION

I have read and understand this consent form, and I volunteer to participate in this research study. I understand that I will receive a copy of this form. I voluntarily choose to participate, but I understand that my consent does not take away any legal rights in the case of negligence or other legal fault of anyone who is involved in this study. I further understand that nothing in this consent form is intended to replace any applicable Federal, state, or local laws.

Participant’s Name (Printed or Typed):
Date:

Participant’s Signature or thumb impression:
Date:

Witness's Signature:
Date:

Signature of Person Obtaining Consent:
Date:
