# Supplementary material for: Relationship Between Staphylococcus aureus Carriage and Surgical Site Infections Following Total Hip and Knee Arthroplasty in the South Asian Population: Protocol for a Prospective Cohort Study
Source: JMIR Res Protoc. 2018 Jun 6;7(6):e10219. doi: 10.2196/10219 (PMC6283255; doi:10.2196/10219)
Supplement: Multimedia Appendix 2 [file resprot_v7i6e10219_app2.pdf]

PERFORMA

Pre-operative data (At the time of sample collection)

Patient MR: ___________ Age: __________ Gender: Male / Female

Height: ____________ Weight: __________________

Comorbidities: HTN (Y/N) DM: (Y/N)
Osteoporosis: (Y/N) Rheumatoid arthritis: (Y/N)
Malignancy: (Y/N) Renal insufficiency: (Y/N)
Peritoneal dialysis/Renal failure: (Y/N) Immunodeficiency: (Y/N) Liver failure: (Y/N)
Skin disease: (Y/N) ____________ Others:_________________________

Previous antibiotic therapy within last one month of current admission:
- name of antibiotic _________________________________
- diagnosis for which antibiotic taken _______________
- duration of antibiotic ______________________________

Previous hospitalizations within the last 6 months
- diagnosis __________________________________
- name of hospital ________________
- time elapsed between previous and current hospital admission ____________

Surgical procedure: __________________________ Side: right / left / bilateral

Date of surgery: ____________________ Surgeon: ______________

ASA: Type of Anesthesia: ____________________

Duration of surgery: _______________ Implant used: _______________

Antibiotic prophylaxis for index surgery:
- Name of antibiotic __________________
- Duration of antibiotic _______________

Post operative data (2nd review)

Pooled swab specimen result: ______________________________

Colony count: ____

SSI: yes / no Date of SSI: _______________________________

Date of discharge: __________________________________

Duration of Hospital stay: _______Days

Re-hospitalized for SSI: ____

Any surgical procedure for SSI: __________________________________

Complications (intraoperative):

(post-operative): SSI/ DVT/ Cardiac complications/ UTI/ Pneumonia/ Hematoma/ Other

____

In-hospital mortality: yes/no
