# Supplementary material for: Relationship Between Staphylococcus aureus Carriage and Surgical Site Infections Following Total Hip and Knee Arthroplasty in the South Asian Population: Protocol for a Prospective Cohort Study
Source: JMIR Res Protoc. 2018 Jun 6;7(6):e10219. doi: 10.2196/10219 (PMC6283255; doi:10.2196/10219)
Supplement: Multimedia Appendix 3 [file resprot_v7i6e10219_app3.pdf]

Multimedia Appendix 1: Relative Risk of each dependent variable with a positive carrier status for *S. aureus*.

Dependent Variable	Crude RR[a] (*P* value)			Adjusted RR (*P* value)		
	MSSA[b]	MRSA[c]	Both	MSSA	MRSA	Both
SSI[d] (infected)						
Postoperative length of stay (prolonged)						
Postoperative complications (yes)						
Rehospitalizations due to SSI (yes)						

[a]RR: relative risk.

[b]MSSA: Methicillin sensitive *Staphylococcus aureus*.

[c]MRSA: Methicillin resistant *Staphylococcus aureus*.

[d]SSI: surgical site infection.
