# Supplementary material for: Relationship Between Staphylococcus aureus Carriage and Surgical Site Infections Following Total Hip and Knee Arthroplasty in the South Asian Population: Protocol for a Prospective Cohort Study
Source: JMIR Res Protoc. 2018 Jun 6;7(6):e10219. doi: 10.2196/10219 (PMC6283255; doi:10.2196/10219)
Supplement: Multimedia Appendix 4 [file resprot_v7i6e10219_app4.pdf]

Multimedia Appendix 2: The relationship between a positive carrier status of *S. aureus* and various patient characteristics.

Potential Risk Factors	Crude OR[a] (*P* value)			Adjusted OR (*P* value)		
	MSSA[b]	MRSA[c]	Both	MSSA	MRSA	Both
**Age (years)**						
<60						
≥60						
**Sex**						
Male						
Female						
**Body mass Index (kg/m²)**						
<30						
≥30						
**Comorbidities present**						
No						
Yes						
**Site of operation**						
Hip						
Knee						
**Duration of surgery**						
Normal						
Prolonged						
**ASA[d] status**						
<3						
≥3						
**Hospital admission within 6 months**						
No						
Yes						
**Antibiotic therapy within 1 month of admission**						
No						
Yes						

[a]OR: odds ratio.

[b]MSSA: Methicillin sensitive *Staphylococcus aureus*.

[c]MRSA: Methicillin resistant *Staphylococcus aureus*.

[d]ASA: American Society of Anesthesiology.
